# RBPs: an RNA editor’s choice

**DOI:** 10.3389/fmolb.2024.1454241

**Published:** 2024-08-06

**Authors:** Ivo Fierro-Monti

**Affiliations:** European Molecular Biology Laboratory - European Bioinformatics Institute (EMBL-EBI), Wellcome Trust Genome Campus, Cambridgeshire, United Kingdom

**Keywords:** RNA editing, RNA-binding proteins (RBPs), single amino acid variants (SAAV), missense variants, proteogenomics, recoding, proteomics

## Abstract

RNA-binding proteins (RBPs) play a key role in gene expression and post-transcriptional RNA regulation. As integral components of ribonucleoprotein complexes, RBPs are susceptible to genomic and RNA Editing derived amino acid substitutions, impacting functional interactions. This article explores the prevalent RNA Editing of RBPs, unravelling the complex interplay between RBPs and RNA Editing events. Emphasis is placed on their influence on single amino acid variants (SAAVs) and implications for disease development. The role of Proteogenomics in identifying SAAVs is briefly discussed, offering insights into the RBP landscape. RNA Editing within RBPs emerges as a promising target for precision medicine, reshaping our understanding of genetic and epigenetic variations in health and disease.

## 1 The ubiquitous role of RBPs in gene expression regulation

RNA-binding proteins (RBPs) are integral to gene expression regulation. Defined by their ability to bind RNA, many of them through modular RNA-binding domains (RBDs), they also contain conserved structural motifs enhancing RNA specificity and functional versatility. About 14%–32% of human protein-coding genes encode RBPs (Baltimore and Huang, 1970; Lunde et al., 2007; Cook et al., 2011; Mallam et al., 2019; Piovesan et al., 2019; Caudron-Herger et al., 2021). RBPs can be classified based on four interaction categories: RNA motif-dependent, RNA structure-dependent, RNA modification-dependent, and RNA guide-based interactions (Liu et al., 2020). These interactions form ribonucleoprotein (RNP) complexes, modulated by RNA through riboregulation, where RNA controls protein function by direct, specific binding.

Dysregulated RBPs are linked to diseases such as cardiovascular and peripheral vascular diseases, diabetes, cancer, neurodegenerative diseases, and autoimmune disorders (Gebauer et al., 2021; Kelaini et al., 2021; Hashimoto and Kishimoto, 2022; Liu and Cao, 2023; Naskar et al., 2023; Sanya et al., 2023). RBPs recognise and interact with numerous transcripts via RBDs, forming regulatory networks essential for controlling protein expression and maintaining cellular homeostasis. Acting as intermediaries, RBPs integrate genetic, epigenetic, transcriptional, post-transcriptional, translational, and environmental cues, leading to variations in protein expression among individuals (Glisovic et al., 2008) (Supplementary Figure S1A). Understanding the role of RBPs in these processes is crucial for elucidating mechanisms underlying phenotypic diversity and disease susceptibility.

**FIGURE 1:**
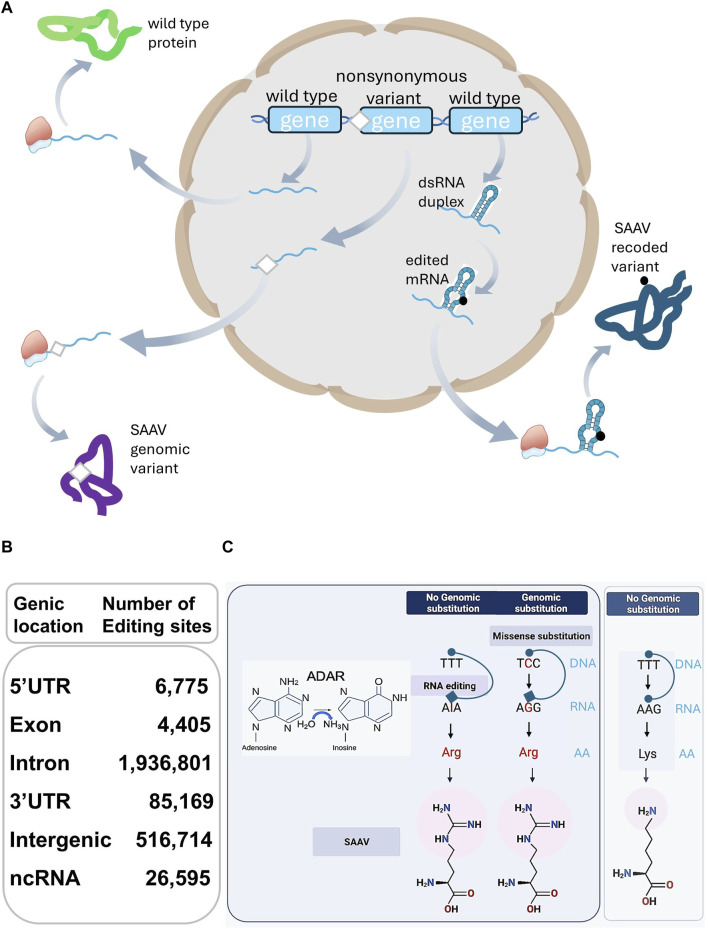
**(A)** SAAVs arise from genomic and RE substitutions, affecting protein sequences and structures differing from their corresponding wild-type counterparts. **(B)** The table describes the total number of human RE sites extracted from the RediPortal database. The total number of sites (2,572,054) present in non-protein coding genomic locations including 5′UTR (6,775), Intron (1,936,801), 3′UTR (85,169), Intergenic (516,714), and non-coding (nc)RNA (26,595) largely surpasses the number of nonsynonymous sites positioned in exons (4,405). **(C)** On the left side of the panel, Adenosine-to-Inosine substitution is catalysed by ADAR hydrolytic deamination activity at the C6 position of adenine. On the right side, a schematic representation of the differences between a genomic missense substitution and an RE-derived single AA substitution. Genomic missense single nucleotide substitution (C-to-G) or an RE event (A-to-I) with no genomic substitution, are both leading to a SAAV, represented as a Lysine-to-Arginine substitution. With no genomic substitution and in the absence of RNA editing, no SAAV but a Lysine is produced (far right side).

Adenosine to Inosine (A-to-I) RNA Editing (RE) is predominantly mediated by double-stranded RNA-binding proteins (dsRBPs) with Adenosine Deaminase Acting on double-stranded (ds)RNA (ADAR) enzymatic activity, catalysing the hydrolytic deamination of adenine at the C6 position (Figure 1C). The most prevalent RE event involves the conversion of A-to-I, with less frequent changes such as cytosine-to-uracil (C-to-U), due to APOBEC1, acting exclusively on single stranded RNA (Bass et al., 1997; Blanc and Davidson, 2010). Specifically, enzymatic activity of ADAR1 and ADAR2, but not ADAR3, are central to this process (Savva et al., 2012), recognising “type A” stem-loop structures via dsRNA binding domains (Ryter and Schultz, 1998), with additional specificity derived from sequence-specific structural features within the dsRNA duplex. Recent studies show that editing-dependent functions of ADAR1 protect dsRNA from dsRNA-sensing molecules causing translation shut down (Chung et al., 2018) and inhibiting innate immunity and the interferon-mediated response. Deficiency in these ADAR1 functions due to 11 mutations affect either the catalytic domain or RNA binding (RB) loops (Wright and Vissel, 2012) and underlie the pathogenesis of autoinflammatory diseases, such as the type I interferonopathies Aicardi-Goutières syndrome.

Unconventional RBPs lacking canonical RBDs, often exhibit context-dependent RB, suggesting influence by cellular conditions or RNA structural features (Sternburg and Karginov, 2020; Van Nostrand et al., 2020; Gebauer et al., 2021; Ray et al., 2023; Sanya et al., 2023). Interacting through non-canonical domains including protein-protein interaction interfaces, enzymatic cores, intrinsically disordered regions (IDRs), these RBPs challenge conventional RNP complex understanding (Garcia-Moreno et al., 2018). RNA-driven interaction mechanisms leading to the formation of RNPs are frequent. This supports the idea that RNA-protein interactions can take place in the absence of classical RBDs in RBPs, emphasising RNA’s regulatory roles as scaffolds, in protein-networks driven cellular processes, or in RNP remodelling mediated by modifications (Lee and Lykke-Andersen, 2013; Garcia-Moreno et al., 2018). The expanding RBP repertoire within the human proteome suggests broader functional interplays and demands a precise and comprehensive catalogue, requiring experimental and computational efforts (Jin et al., 2023).

RBPs enhance network interactions by controlling transcription through regulatory RNAs. Like transcription factors (TFs), RBPs are associated with genome hotspots, particularly gene promoters, influencing transcriptional output (Xiao et al., 2019). Additionally, RBPs mediate post-transcriptional control of gene expression, including splicing, transport, modification, translation, and degradation, often involving novel RBPs like PRRC2B, with an essential role in translation required for cell cycle progression (Jiang et al., 2023). Leaky scanning, where ribosomes bypass upstream Open Reading Frames (uORFs) which slow down the translation of the main ORF, is also regulated by PRRC2 proteins. These RBPs bind translation initiation factors and preinitiation complexes, facilitating translation initiation on mRNAs with uORFs (Bohlen et al., 2023). Many mRNAs with uORFs promote translation reinitiation and bypass uORF-mediated suppression mediated by RBPs such as *Drosophila* Nocte. Nocte RBP is critical for *Drosophila* eye development, with disruptions leading to developmental defects and neurological disorders (Zhang et al., 2024).

**FIGURE 2:**
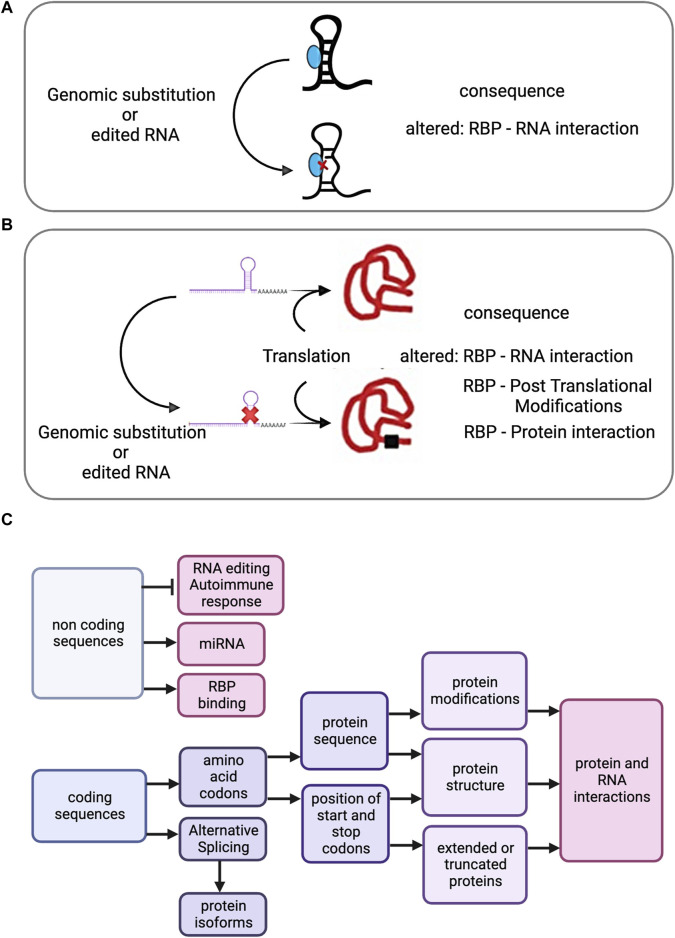
**(A)** Effect of genomic substitution or RNA recoding on Riboregulation. Either genomic substituted derived transcripts or recoded RNAs present in coding or non-coding RNAs may have implications for Riboregulation. A diagram showing a non-coding transcript with a dsRNA region, the recoded or genomic substituted derived transcript and its possible effect on the RNA - RBP interaction. **(B)** After translation, a recoded or genomic substituted derived RBP encoding transcript is represented, as well as the possible effects. **(C)** Cascade of potential effects propagated by SAAVs (derived from genomic or RNA editing substitutions) or by RNA editing recoding events in non-coding regions.

RBPs undergo multifaceted modifications influenced by RE and genomic variants. Alterations affect expression levels, generating distinct isoforms and amino acid (AA) sequence modifications with significant implications, including post-translational modifications (PTMs), altered protein-protein interactions, and subcellular localisation changes (Gebauer et al., 2021) (Figure 2B). While most editing targets untranslated regions (Hundley and BASS, 2010; Ellingford et al., 2022), highlighting the potential changes occurring within coding regions is required, as genomic and RE missense substitutions can have pathogenic effects, supporting the exploration of underlying mechanisms.

This manuscript explores the connection between RBPs, post-transcriptional RE, and resulting Single Amino Acid Variants, SAAVs, influencing health and disease. Sections examine the RBP landscape, emphasising physiological recoding, RE, disease implications, and the evolving field of proteogenomics, offering a holistic perspective and insights from cutting-edge omics studies.

## 2 Genomic and RNA editing effects: exploring the human RNA encoding RBP editome

SAAVs in the proteome arise from the interplay of genomic and nonsynonymous RE missense events (Chen et al., 2020; Petrosino et al., 2021). While distinct, these mechanisms converge, influencing RBPs and impacting phenotypic outcomes (Figure 1A).

In contrast to genomic missense mutations, RE events are sporadic within transcripts encoding small ORFs and even rarer in the broader protein-encoding transcriptome (Picardi et al., 2017), constituting approximately less than 1% of nonsynonymous RE events (Figure 1B). This distinction lies in the preservation of the DNA sequence encoding the target transcripts during RE, in contrast to missense mutations (Bass, 2002), as depicted in Figure 1C.

The protein-coding transcriptome undergoing RE, documented in the REDIportal database (Mansi et al., 2021), includes diverse human RNA sequencing samples from Genotype-Tissue Expression (GTEx) and provides insights into RE prevalence. Beyond REDIportal, other databases (Kiran and Baranov, 2010; Ramaswami and Li, 2014; Mallam et al., 2019; Niu et al., 2019) have delineated physiological and aberrant RE in coding transcripts.

The potential translation of these edits into SAAV events, including missense mutations, at the protein level underscores their significance. The best-studied A-to-I editing substrates are the brain-specific transcripts coding for glutamate receptor (GluR) channels. AMPA-type (α-amino-3-hydroxy-5-methyl-4-isoxazolepropionate) glutamate receptors (AMPARs) are critical for fast excitatory transmission in the central nervous system. A key regulatory step involves RE of the GluA2 subunit transcript by the dsRBP ADAR2 (Lin and Chen, 2019). This editing event recodes glutamine to an arginine codon, altering the encoded AA within the AMPAR heterotetrameric channel pore region. This event is essential for proper AMPAR function, as unedited GluA2 subunits containing glutamine allow calcium influx, while edited subunits with arginine are calcium impermeable. Notably, nearly complete editing occurs at this specific site, highlighting its critical role. Disruption of this editing process, leading to unedited GluA2 subunits, can result in pathological calcium influx, potentially contributing to seizures and neuronal cell death (Lin and Chen, 2019).

High-throughput techniques, relying on UV-crosslinking, affinity purification, size-exclusion chromatography, and mass spectrometry (MS)-based proteomics, have identified RBPs, expanding the RBPome, a term encompassing the entire repertoire of RBPs (Sommer et al., 1991; Nechay and Kleiner, 2020; Gebauer et al., 2021). As methodologies evolve, elusive proteins with weak crosslinking or low expression become accessible. The surge in RBPs leads to diverse studies and databases (RBPbase (Gebauer et al., 2021), Mallam et al.’s database (Mallam et al., 2019), RBP2go (Caudron-Herger et al., 2021), EuRBPdb (Liao et al., 2020), RBPDB (Cook et al., 2011), RNAcompete (Ray et al., 2009; Van Nostrand et al., 2020)), highlighting the evolving RBP landscape.

A human superset of 2,650 RBPs, identified in at least two RNA interaction capture (RIC) studies, combined with curated lists from Gene Ontology (The Gene Ontology, 2019), RBPDB, and RNAcompete, serves as the comprehensive RBPbase resource - “humanRBPs-2021 RBPANNO000000078.1” - consisting of 3,470 RBPs, and here designated “humanRBPs-2021.” This comprehensive RBPbase resource (Resource 2) assists as a powerful tool to pinpoint RBPs implicated in genetic diseases (Gebauer et al., 2021) and it was selected as a basis for the analysis of RE events in RBPs, as it represents a combination identified in multiple high-throughput studies and a set of well-known and defined RBPs.

Leveraging editing events from REDIportal - a rich repository of human physiological RE events - reveals a substantial “human coding editome” defined by a pooled editing level exceeding 1% (2,414 editable proteins, Resource 1), where around one-fifth represents potentially recoded RBPs with RE events propagated to their AA sequences (hypergeometric test, p 6.6e-7, corrected for multiple testing), a previously unappreciated protein class target within the human protein-coding editome (Supplementary Figure S2A). This suggests a mechanism where RBP-encoding RNA undergo editing, propagating SAAVs diversifying the proteome, and impacting their function, localisation, and interactions (Eisenberg, 2021). Approximately 12% of the human protein-encoding genome, editable RBPs devoid of known RBDs, may engage with RNA through unconventional means, emphasising the importance of considering editable RNA-determined interactions in RNP formation (Beckmann et al., 2016). Approximately 3.4%, comparably lesser RBPs with RBD-Pfam domains are frequently editable, suggesting a higher functional diversity beyond RB. Statistical tests indicate a significant enrichment of gene ontology term “Ribosome” and protein classes such as “Ribosomal protein” and “translational protein” among editable RBPs (*p* < 0.05, corrected for multiple testing), hinting at a potential link to translational control (Supplementary Figure S2B).

## 3 Implications of RNA editing in disease and development

RE primarily occurs in non-coding regions, with intronic transcripts more frequently modified than intergenic counterparts (Figure 1B). A-to-I conversion by ADAR, marks dsRNA as “self,” preventing unsuitable immune responses (Lamers et al., 2019). ADAR safeguards against autoimmunity by suppressing pathways associated with cellular long dsRNA sensors, including antiviral RIG-I-like receptors, MDA5-MAVS pathway, Protein Kinase activated by RNA (PKR), and OAS-RNAses (Quinones-Valdez et al., 2019). ADAR-interacting RBPs regulate A-to-I editing (Reich and Bass, 2019). Without ADARs, long dsRNAs may be mistaken for viral dsRNA, eliciting abnormal immune responses (Shtrichman et al., 2012).

RE plays key roles in various biological processes, including embryonic development (Shtrichman et al., 2012), neuron generation (Greger et al., 2003), and somatic cell reprogramming (Guallar et al., 2020). The phenomenon of RNA recoding can be transient during early development, with varying tissue-specific levels of RE. Single-cell RNA sequencing analyses established dynamic changes in ADAR expression and stage-specific RE during early embryogenesis (Qiu et al., 2016). Spatiotemporal expression patterns of ADAR1 and ADAR2 examined during the development of mouse forebrain disclosed a broad distribution in most regions, with their precise colocalisation in neurons, and uncovered that editing for specific ADAR mRNA targets precede high levels of expression maintained into adulthood (Jacobs et al., 2009). Based on RNA sequencing, the transcriptional profiles of cloned and fertilised bovine embryos were compared. Cloned embryos were shown to lack RE sites, which may have resulted from a decreased ADAR expression. Consequently, the authors concluded that cloned embryo development may be affected by decreased ADAR expression and incomplete RE, and their analysis provided new data for further mechanistic studies of somatic nuclear reprogramming (Zhang et al., 2020).

Dysregulation of RE can trigger immune responses by forming dsRNA duplexes, potentially leading to autoimmune disorders like psoriasis, rheumatoid arthritis, systemic lupus erythematosus, and multiple sclerosis (Li et al., 2022). The human brain and cardiovascular tissues exhibit a high prevalence of RE events (Ma et al., 2021; Mann et al., 2023), with 30% of recoded transcripts encoding RBPs (“humanRBPs-2021”) such as COPA, PUM2, PDC7, SON, RHOA, RRNAD1, SRP9, PPIL3, FLNA, TSEN2, and NOP14, linking RE of RBPs to cardiovascular diseases (Chen et al., 2020).

Non-coding miRNA recoding affects miRNA binding and translation control, with dysregulated expression of recoded ncRNAs being a feature of cancer cells and the malignant microenvironment (Liu et al., 2014; Torsin et al., 2021). Large-scale analysis of A-to-I and C-to-U editing in human miRNAs across 13 tissues revealed hypo-editing of miRNA and downregulation of ADAR2 in glioblastoma samples (Paz-Yaacov et al., 2015). In osteosarcoma, up-regulated editing sites in the 3′UTR abolish miRNA binding, increasing the expression of EMP2 and several oncogenes, highlighting miRNA editing’s role in oncogenesis (Ge et al., 2023). A study of melanoma patients treated with immunotherapy found 34% of RE sites in the 3′UTR targeted miRNAs, and 66% in coding regions were non-synonymous mutations leading to AA substitutions (Lu et al., 2024).

## 4 Significance of proteogenomics in uncovering SAAVs and assessing alterations in the recoded proteome

Proteogenomics integrates DNA/RNA sequencing with MS-based proteomics to reveal frequently recoded human proteins, including ubiquitously expressed RBPs (Gabay et al., 2022; Levitsky et al., 2023). This approach enhances understanding of genomic and RE missense substitutions, and identifies unannotated non-canonical variants, driver genes, and novel therapeutic targets across cancers (Li et al., 2023; Liao et al., 2023; Wang et al., 2023). Advances in omics technologies, supported by AI-assisted bioinformatic tools, improve the identification of SAAVs and sequence alignment at RNA and protein levels (De Paoli-Iseppi et al., 2021; Dai and Shen, 2022). Deep learning models predict protein structures and assess missense substitutions’ pathogenicity, enhancing protein stability predictions based on SAAV data (Biswas and Chakrabarti, 2020; Alquraishi, 2021; Hagg and Kirschner, 2023; Jagota et al., 2023).

MS-based proteomics datasets show enrichment of A-to-I nonsynonymous RE events at PTM sites, such as acetylation, methylation, sumoylation, and ubiquitination, affecting protein functions like degradation and subcellular localisation (Li et al., 2022). Changes in PTMs within RBPs, identified through proteogenomics, may alter RNP complexes and interactomes (Figure 2B). Proteogenomics pinpoints SAAVs, while subsequent proteomics explores changes within the recoded proteome including PTMs and interconnected networks. These advancements deepen our understanding of genetic and epigenetic variations, enabling biomarker discovery and advancing precision medicine.

## 5 RNA editing, RBPs, and disease: a complex frontier

The complex relationship between RE and its impact on RBPs in disease development deserves comprehensive exploration.

RE is primarily catalysed by dsRBP ADAR1 (and ADAR2), and SAAVs stemming from genomic missense mutations are associated with rare genetic pigmentation disorders and autoimmune diseases, such as Dyschromatosis symmetrica hereditaria and Aicardi-Goutières syndrome (Wright and Vissel, 2012). In addition, recent data from REDIportal reports A-to-I editing of the human dsRBP ADAR transcript, yielding potential SAAVs within the human ADAR protein (Supplementary Figure S1B). *Drosophila* ADAR2 transcript likewise undergoes RE (Keegan et al., 2005), where serine is substituted by glycine near the deaminase active site. The level of this Adar S-to-G self-editing is low in embryos and increases to 40% in adult flies. The edited ADAR G isoform is less active than the genome-encoded ADAR S isoform, both *in vivo* and *in vitro* (Keegan et al., 2023).

Elevated A-to-I editing levels, mediated by ADAR1 and ADAR2 enzymes, significantly impact cancer (Zipeto et al., 2016; Jiang et al., 2019). ADAR1, a “double-edged sword”, promotes leukemic stem cell proliferation by influencing let-7 microRNA biogenesis and activating the JAK2 signalling pathway, highlighting its potential as a therapeutic target in leukaemia (Zhang and Slack, 2016). It also hyperedits cell-cycle regulatory and tumour suppressor mRNA, further promoting leukaemia development (Zhang and Slack, 2016). Conversely, reduced A-to-I editing, often involving ADAR2 downregulation, inhibits astrocytoma migration and proliferation (Cenci et al., 2008). This tumour-suppressive role also extends to oesophageal squamous cell carcinoma, where ADAR2 editing of IGFBP7 mRNA disrupts tumorigenic pathways.

### 5.1 Editing of RNA, RNA-RBP interactions and membrane-less organelles

Riboregulation signifies a paradigm shift in our understanding of RNA-RBP interactions, with RNA emerging as a regulator of RBP function (Hentze et al., 2018). This concept challenges the traditional view of RBPs as primary regulators of RNA, emphasising their reciprocal interplay. Examples include metabolic enzymes (Huppertz et al., 2022), dsRNA-binding biosensor proteins like PKR (Gal-Ben-Ari et al., 2018), and antiviral receptors such as PRR and MDA5 (Quinones-Valdez et al., 2019). RE, especially targeting non-coding region transcripts, or RBP-recoded variants can modulate RNA-RBP interactions, either amplifying or diminishing their regulatory effects (Figures 2A, B). A report surveyed the binding preferences of 150 RBPs to RE events, focusing on A-to-I editing in two human cell lines. It was deduced that changes in RE could alter RNA secondary structures, affecting RBP-binding preferences and influencing post-transcriptional processes like RNA splicing, structure formation, and decay (Hu et al., 2022).

RBPs possess IDRs (Huai et al., 2022), making them vulnerable to changes induced by genomic missense or RNA RE variants. These alterations can affect RBPs’ ability to undergo liquid-liquid phase separation (LLPS), crucial for forming dynamic RNP networks or membrane-less organelles (MLOs) like nucleoli, stress granules, P-bodies, and nuclear speckles. LLPS is essential for various cellular processes, including transcription, translation, and signal transduction (Tsang et al., 2020; Li et al., 2023; Dai and Yang, 2024). For example, nuclear speckles regulate gene expression by storing and modifying pre-mRNA splicing factors (Hofmann et al., 2021; King et al., 2024).

SON, an RBP, localises to nuclear speckles, which regulate gene expression by storing and modifying pre-mRNA splicing factors (Supplementary Figure S1B) (Kim et al., 2019; Ilik et al., 2020). SON acts as an mRNA splicing cofactor, facilitating the efficient splicing of numerous cell-cycle and DNA-repair transcripts. REDIportal has catalogued forty RE events in transcripts encoding SON RBP, with a notable increase of over 10% in bladder urothelial carcinoma compared to healthy tissue (Gabay et al., 2022). Of these forty substitutions, nineteen are within IDRs, some potentially having pathogenic implications.

Cross-referencing healthy editable RBPs from the RBPbase “humanRBPs-2021” with an MLO dataset (5,282 genes) reveals that approximately two-thirds of these RBPs may inhabit MLOs (p 1.2e-80) (documented in MLOsMetaDB - http://mlos.leloir.org.ar) (Supplementary Figure S2C). Disrupted LLPS is linked to various pathological conditions, including cancer, viral infections, and neurodegenerative disorders (Huai et al., 2022). Recoded RBPs within MLOs can remodel interactomes through PTMs and protein-protein interactions, influencing LLPS dynamics. For instance, multiple PTMs of FUS, such as serine and threonine phosphorylation and arginine methylation, significantly impact its aggregation and LLPS properties (Qamar et al., 2018).

### 5.2 Additional examples of prevalent recoded RBPs COPA, FLNA, and FLNB uncovered in recent proteogenomic analyses

The recoded COPA RBP (Supplementary Figure S1B) at position I164V within a WD40 repeat affects protein interactions and is highly expressed in diseases like bladder urothelial carcinoma, breast cancer, and liver adenocarcinoma (Slotkin and Nishikura, 2013; Lundin et al., 2020; Gabay et al., 2022). COPA is involved in transporting dilysine-tagged proteins from the endoplasmic reticulum (ER) to the Golgi apparatus. Its recoding may cause ER stress, contributing to unfolded protein responses seen in various diseases, including cancer, neurodegeneration, diabetes, and inflammatory disorders.

Recoding of the FLNA RBP (Supplementary Figure S1B), specifically glutamine to arginine exchange, regulates angiogenesis in tumours. Hyper-editing reduces angiogenesis, while hypo-editing enhances it, potentially by altering VEGFR2 turnover. This modification also influences tumour metastasis through extracellular matrix interactions, highlighting FLNA editing’s role in angiogenesis, tumour growth, and metastasis (Jain et al., 2022).

FLNB RBP (Supplementary Figure S1B) recoding, specifically the M2293V edit, appears to suppress the growth- and invasion-inhibiting functions of the protein by affecting its nuclear localisation, thereby diminishing its EMT-suppressive role. This recoding likely disrupts FLNB RBP function by interfering with binding partners, impacting both its function and localisation (Levitsky et al., 2023).

### 5.3 Frequent RBP-derived neoantigens identified via immunopeptidomics

In a noteworthy study involving cancer patients, the integration of whole exome sequencing (WES), RNA sequencing (RNA-seq), and MS-based Immunopeptidomics revealed predominant RNA-derived neoantigens originating from coding regions, with approximately one-third of protein-coding genes exhibiting missense substitutions correlating with the RBPbase database “humanRBPs-2021.” Immunopeptidomics-driven identification of tumour neoantigens holds promise for personalised anti-cancer vaccines, making far-reaching changes in treatment strategies based on individual genomic and proteomic landscapes (Tretter et al., 2023; Zhang and Bassani-Sternberg, 2023).

### 5.4 Role of RE in disease and therapeutic potential

Genetic variants linked to RE, i.e., edQTL analyses, underscore the role of RNA secondary structure in dictating RE levels at specific sites, impacting complex traits and diseases by modulating the stability and levels of crucial RNA molecules (Park et al., 2021). mRNA editing’s involvement in various diseases, such as glioblastomas, inflammatory and autoimmune disorders, and its positive correlation with somatic point mutation burden in cancer, including the development of a novel metric, RE load, underscores its significance (Paz-Yaacov et al., 2015; Paul et al., 2017; Baker et al., 2022).

The impact of cellular stress-induced hyper-editing extends beyond cancer to cardiovascular diseases, highlighting mRNA recoding’s role in diverse pathologies (Mann et al., 2023). These collective findings expand our understanding of the interplay of genetic and epigenetic variations in health and disease, potentially catalysing a paradigm shift in medicine. RE emerges as a therapeutic target across a spectrum of diseases, showcasing its versatility in modulating life-threatening pathologies (Khosravi and Jantsch, 2021; Booth et al., 2023).

One potential RE therapeutic approach to rectifying G-to-A nonsense mutations involves the utilisation of endogenous ADAR. For instance, in scenarios involving a TAG stop codon disease variant, a guide RNA can be engineered to complement the mutated region, recruiting ADAR to edit the mRNA UAG stop codon back to UGG. This corrective action effectively eradicates the nonsense mutation, allowing mRNA translation to proceed unimpeded. These transformative developments highlight the profound influence of RE in shaping the landscape of genetic and epigenetic variation, offering new avenues for precision medicine tailored to individual patient’s unique genetic profiles (Pfeiffer and Stafforst, 2023).

## 6 Concluding remarks

The relationship between RE, RBPs, and disease unveils a significant area for research and therapeutic innovation. Understanding this interplay will enhance our comprehension of genetic and epigenetic variations underlying health and disease.

Proteogenomics, by elucidating RE events and identifying SAAVs in RBPs, serves as a potent tool for discovering disease biomarkers and developing tailored diagnostics and treatments. While the precise mechanisms of RE variants influencing RBPs in disease remain unclear, further investigation will clarify the complex interactions between edited and unedited dsRNAs and RBPs.

Deciphering the impact of RBP RE variants on key RNAs in disease mechanisms promises to provide biomarkers and therapeutic insights. Actively researching these interactions is crucial for understanding disease mechanisms and developing novel therapies.

RE emerges as a target for interventions across various life-threatening conditions, extending beyond cancer to a broader range of diseases. This highlights RE’s potential to advance precision medicine and enable personalised treatments, significantly transforming healthcare.
