# Supplementary material for: RBPs: an RNA editor’s choice
Source: Front Mol Biosci. 2024 Aug 6;11:1454241. doi: 10.3389/fmolb.2024.1454241 (PMC11333368; doi:10.3389/fmolb.2024.1454241)
Supplement: Supplementary file 1 [file DataSheet1.ZIP › Supplementary data/Supplementary data/Supplementary Document.docx]

B


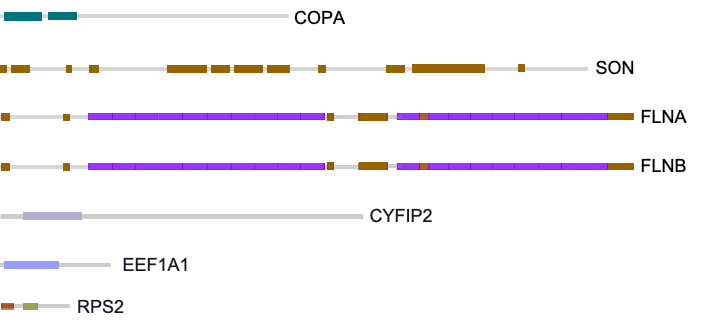


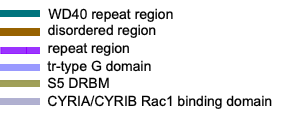


**Supplementary Figure 1A.** RBPs occupy a pivotal position in coordinating regulatory networks governing gene expression. Functioning as mediators, RBPs integrate a myriad of signals encompassing genetic, epigenetic, transcriptional, post-transcriptional, translational, and environmental cues. Given the indispensable role of RBPs in these regulatory cascades, genomic substitutions within the coding regions of RBP genes and their corresponding transcripts hold the potential to exert profound influences on subsequent biological processes. Such changes, in turn, may contribute to discernible phenotypes that extend beyond individual variations, embodying signs of both health and disease. The global impact of such genomic substitutions and RNA editing events in RBPs is instrumental in shaping the phenotypic landscape across diverse individuals. **1B.** Selection of prevalent recoded RBPs identified in recent proteogenomic studies. Diverse structural domains present in these RBPs (unedited form), with disordered regions as common features in four of them.

**Supplementary Figure 2A.** Venn diagram representing the intersections between the RNA edited transcripts encoding RBPs extracted from RediPortal and the RBPs from the “humanRBPs-2021” considering as a reference the human protein encoding genome. Number of genes and percentages are shown for each category. A hypergeometric test indicates the cross-reference set of editable RBPs is statistically significant with a p value 6.6e-7. **2B.** Statistical overrepresentation test performed on the editable RBP set versus the humanRBPs-2021 as a reference, using the Panther database (https://www.pantherdb.org/). In the editable RBP set, significantly overrepresented terms (Bonferroni corrected, p < 0.05) were Protein Class ‘Ribosomal Protein’ and ‘Translational Protein’, as described in the Gene Count plot. No statistically significant Protein Class terms were identified when the non-editable RBP set was tested using the humanRBPs-2021 reference set. **2C.** MLOs and RBPs. Venn diagram representing the intersection between the editable RBPs and the Membrane-less Liquid Organelles MLO’s genes dataset (documented in MLOsMetaDB - http://mlos.leloir.org.ar) considering as a reference the human protein encoding genome. Number of genes and percentages are shown for each category. A hypergeometric test indicates the cross-reference set of editable RBPs and MLO gene set is statistically significant with a p value 1.2e-80.
